# CXCL12 is expressed by skeletal muscle cells in tongue oral squamous cell carcinoma

**DOI:** 10.1002/cam4.5392

**Published:** 2022-10-27

**Authors:** Akira Yorozu, Shohei Sekiguchi, Akira Takasawa, Fumika Okazaki, Takeshi Niinuma, Hiroshi Kitajima, Eiichiro Yamamoto, Masahiro Kai, Mutsumi Toyota, Yui Hatanaka, Koyo Nishiyama, Kazuhiro Ogi, Hironari Dehari, Kazufumi Obata, Makoto Kurose, Atsushi Kondo, Makoto Osanai, Akihiro Miyazaki, Kenichi Takano, Hiromu Suzuki

**Affiliations:** ^1^ Department of Otolaryngology‐Head and Neck Surgery Sapporo Medical University School of Medicine Sapporo Japan; ^2^ Department of Molecular Biology Sapporo Medical University School of Medicine Sapporo Japan; ^3^ Department of Oral Surgery Sapporo Medical University School of Medicine Sapporo Japan; ^4^ Department of Pathology Sapporo Medical University School of Medicine Sapporo Japan

**Keywords:** CXCL12, muscle cells, OSCC, prognosis

## Abstract

**Background:**

The CXCL12/CXCR4 axis plays a pivotal role in the progression of various malignancies, including oral squamous cell carcinoma (OSCC). In this study, we aimed to clarify the biological and clinical significance of CXCL12 in the tumor microenvironment of OSCCs.

**Methods:**

Publicly available single‐cell RNA‐sequencing (RNA‐seq) datasets were used to analyze CXCL12 expression in head and neck squamous cell carcinomas (HNSCC). Immunohistochemical analysis of CXCL12, α‐smooth muscle antigen (α‐SMA), fibroblast activation protein (FAP) and CD8 was performed in a series of 47 surgically resected primary tongue OSCCs. Human skeletal muscle cells were co‐cultured with or without OSCC cells, after which CXCL12 expression was analyzed using quantitative reverse‐transcription PCR.

**Results:**

Analysis of the RNA‐seq data suggested CXCL12 is abundantly expressed in stromal cells within HNSCC tissue. Immunohistochemical analysis showed that in grade 1 primary OSCCs, CXCL12 is expressed in both tumor cells and muscle cells. By contrast, grade 3 tumors were characterized by disruption of muscle structure and reduced CXCL12 expression. Quantitative analysis of CXCL12‐positive areas within tumors revealed that reduced CXCL12 expression correlated with poorer overall survival. Levels of CXCL12 expression tended to inversely correlate α‐SMA expression and positively correlate with infiltration by CD8+ lymphocytes, though these relations did not reach statistical significance. CXCL12 was significantly upregulated in muscle cells co‐cultured with OSCC cells.

**Conclusion:**

Our results suggest that tongue OSCC cells activate CXCL12 expression in muscle cells, which may contribute to tumor progression. However, CXCL12 is reduced in advanced OSCCs due to muscle tissue destruction.

## Graphical abstract

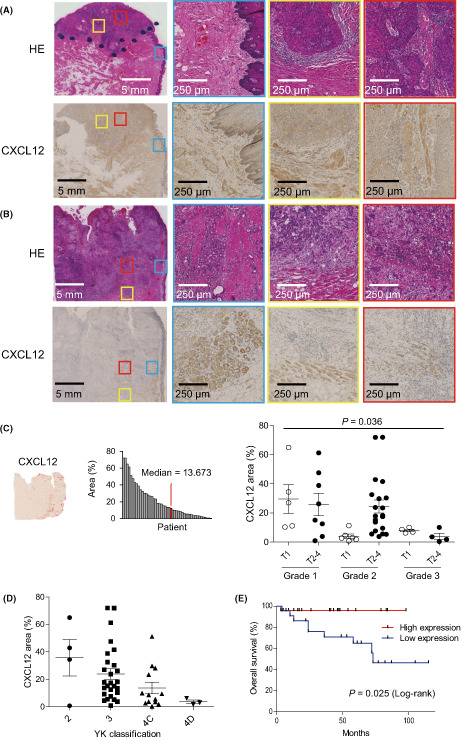

## 1 INTRODUCTION

Initially identified in bone marrow stromal cells, the cytokine CXCL12—also known as stromal cell‐derived factor‐1 (SDF‐1α)—is widely expressed in human tissues, including connective tissue, liver, muscle, bone marrow, and lymph nodes. Its functional receptor, CXCR4, is highly expressed in various human malignancies, and numerous studies have shown that the CXCL12/CXCR4 axis promotes tumor growth, angiogenesis, invasion, and metastasis.^1^ It has also been reported that CXCL12 is secreted from cancer‐associated fibroblasts (CAFs) and that it plays an immunosuppressive role in the tumor microenvironment.^2^, ^3^, ^4^

Head and neck squamous cell carcinomas (HNSCCs) are the most common malignancies arising in the head and neck.^5^ Investigations into the biological and clinical significance of the CXCL12/CXCR4 axis in HNSCCs have found that CXCL12 promotes HNSCC cell invasiveness by activating NF‐κB signaling.^6^ In addition, multiple studies have shown that CXCR4 expression is frequently upregulated in oral squamous cell carcinomas (OSCCs) and that high CXCR4 expression is associated with lymph node metastasis, tumor recurrence, and a poor prognosis.^7^, ^8^, ^9^, ^10^ Similar observations were reported for laryngeal as well as hypopharyngeal squamous cell carcinomas, where CXCL12 is expressed in both stromal cells and lymphocytes within the hypopharyngeal tumor tissues.^11^ Expression of CXCL12 in HNSCC cells is controversial. For instance, two groups reported that they did not detect CXCL12 in HNSCC cell lines tested.^7^, ^11^ In contrast, Zhang et al. recently reported that CXCL12 is frequently expressed in OSCC cells and that its expression is associated with poor differentiation, advanced stage, tumor recurrence, poor survival, and tumor infiltration by Foxp3+ lymphocytes.^12^ It has also been reported that serum CXCL12 levels are higher in HNSCC patients than in healthy individuals, though the higher levels are not associated with tumor size, lymph node metastasis, or tumor stage.^13^

The results summarized above suggest CXCL12 plays an important role in the development and progression of HNSCC, though its expression in the microenvironment of HNSCC is not fully understood. In the present study, we aimed to clarify the biological and clinical importance of CXCL12 in tongue OSCC through analysis of its protein expression in surgically resected OSCC tissues.

## 2 MATERIALS AND METHODS

### 2.1 Transcriptome data analysis

Single‐cell RNA‐sequencing (RNA‐seq) data from primary and metastatic HNSCC tissues reported by Puram et al.^14^ and bulk RNA‐seq data from The Cancer Genome Atlas (TCGA) were obtained from UCSC Xena (https://xena.ucsc.edu/). The data were analyzed and visualized using GraphPad Prism 5 (GraphPad Software).

### 2.2 Tissue samples and cell culture

Primary tongue OSCC tissues were collected from Japanese patients (*n* = 47) who underwent surgical resection at Sapporo Medical University Hospital between April 2009 and April 2019. Two patients received chemotherapy, and three patients received chemotherapy and radiotherapy prior to the surgical treatment. Informed consent was obtained from all patients before collection of the specimens. This study was approved by the Institutional Review Board at Sapporo Medical University (No. 322–38). TNM stages were evaluated according to the eighth edition of the American Joint Committee Caner (AJCC) staging system. The SAS tongue OSCC cell line was cultured as described previously.^15^ Human skeletal muscle myoblasts (HSMMs) were purchased from Lonza (Basel, Switzerland) and cultured in SKBM‐2 Skeletal Muscle Cell Growth Basal Medium‐2 (Lonza) with SKGM‐2 Skeletal Muscle Cell Growth Medium‐2 SingleQuots Supplements and Growth Factors (Lonza). Human skeletal muscle cells were purchased from Takara Bio Inc. (Kusatsu, Japan) and cultured using a skeletal muscle cell growth medium kit (Takara Bio Inc.). For co‐culture experiments, HSMMs (1.5 × 10^5^ cells in 6‐well plates) were indirectly co‐cultured with or without SAS (1.2 × 10^6^ cells in culture inserts) for 8 days until they differentiated into muscle cells. SkMCs (1.5 × 10^5^ cells in 6‐well pates) were co‐cultured with or without SAS (1.0 × 10^6^ cells in culture inserts) for 48 or 96 h. Where indicated, HSMMs were treated for 8 days with 1 or 100 ng/mL transforming growth factor‐β1 (TGF‐β1; PeproTech).

### 2.3 Immunohistochemistry

Immunohistochemical staining was carried out as described previously.^16^ A rabbit anti‐SDF1 (CXCL12) polyclonal Ab (1:200 dilution, ab9797; Abcam), mouse anti‐α‐smooth muscle antigen (α‐SMA) monoclonal Ab (1:50 dilution, M0851; CiteAb) and mouse anti‐CD8 monoclonal Ab (prediluted, clone C8/144B; DAKO) were used. We evaluated the correlation between CXCL12 expression and OSCC histological grades based on the World Health Organization (WHO) and YK classifications.^17^, ^18^ Immunohistochemistry was performed and evaluated by pathologists (Akira Takasawa and Makoto Osanai). To accurately quantitate CXCL12 expression levels over entire tumor areas, we measured the positively stained areas using ImageJ software (NIH) as described previously.^19^ Muscle cell areas were quantified using ImageJ software.

### 2.4 Quantitative reverse transcription‐PCR

Single‐strand cDNA preparation and quantitative reverse transcription‐PCR (qRT‐PCR) were carried out as described previously.^16^ The primer sequences for CXCL12 were 5′‐ AGAGCCAACGTCAAGCATCT‐3′ and 5′‐ AATCCACTTTAGCTTCGGGTC‐3′; those for CXCR4 were 5′‐ACTGAGAAGCATGACGGACAA‐3′ and 5′‐GTTCCCAAAGTACCAGTTTGC‐3′; and those for β‐actin (ACTB) were 5′‐GCCAACCGCGAGAAGATGA‐3′ and 5′‐AGCACAGCCTGGATAGCAAC‐3′.

### 2.5 Statistical analysis

Fisher's exact test was used for the analysis of categorical data. Quantitative variables were analyzed using Student's *t* tests or ANOVA with post hoc tests. Survival was analyzed using the Kaplan–Meier method. Survival curves were compared using the log‐rank test for 2‐group comparisons. Values of *p* less than 0.05 (2‐sided) were considered statistically significant. Statistical analyses were carried out using GraphPad Prism 5 (GraphPad Software).

## 3 RESULTS

### 3.1 CXCL12 is expressed in stromal cells within HNSCC tissues

**FIGURE 1:**
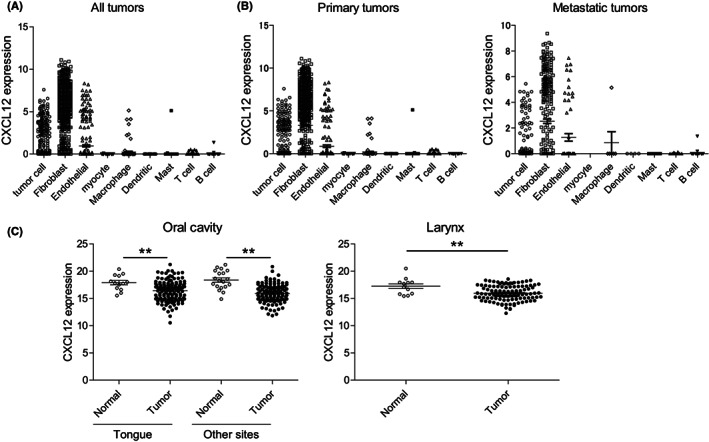
Expression levels of CXCL12 mRNA in primary HNSCC tumors. (A) Summarized results of single‐cell RNA‐seq showing levels of CXCL12 expression in the indicated cell types obtained from HNSCC tissues (Puram et al., Cell, 2017). (B) Results in (A) are segregated into those obtained from primary (left) and metastatic (right) HNSCCs. (C) Summarized results of TCGA RNA‐seq showing levels of CXCL12 expression in HNSCC tumors and normal tissues in the oral cavity and larynx. Error bars represent standard errors of mean (SEMs).

To assess the pattern of CXCL12 expression in HNSCC, we first analyzed publicly available single‐cell RNA‐seq data obtained from primary and metastatic HNSCC samples.^14^ We found that CXCL12 is abundantly expressed in fibroblasts within HNSCC tumors, while lower levels of CXCL12 expression were detected in the tumor cells and endothelial cells (Figure 1A). When samples were divided into primary and metastatic tumors, expression patterns were similar in the two groups (Figure 1B). In addition, RNA‐seq data from primary HNSCC in TCGA datasets revealed that levels of CXCL12 expression are lower in tumors than normal tissues in both the oral cavity and larynx (Figure 1C). These data suggest that CXCL12 is expressed in stromal cells within HNSCC tissue and that it is downregulated as compared to levels in corresponding normal tissues.

### 3.2 CXCL12 expression is downregulated in high grade OSCC

**FIGURE 2:**
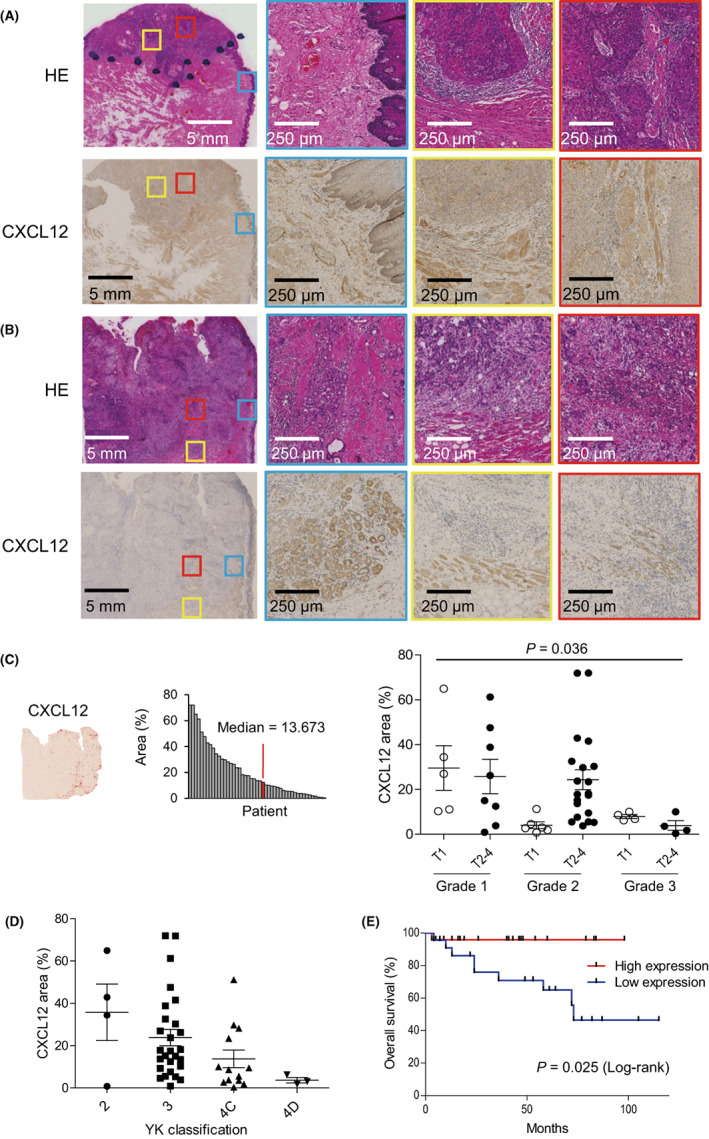
Analysis of CXCL12 expression in primary OSCC tissues. (A, B) Hematoxylin and eosin (HE) staining and immunohistochemical detection of CXCL12 in representative grade 1 (A) and grade 3 (B) OSCC tissues. Magnified views of adjacent normal tissues (blue), invasive front regions (yellow), and central tumor areas (red) are also shown. (C) Areas positive for CXCL12 in a representative tumor are shown on the left. Summarized results for CXCL12‐positive areas in OSCC tissues (*n* = 47) are shown in the center. The extents of CXCL12‐positive areas within OSCC tissues with indicated staging are shown on the right. (D) Areas positive for CXCL12 in OSCC tissues with the indicated YK classification. (E) Kaplan–Meier curves showing the association between CXCL12 expression and survival among patients with OSCC.

We used immunohistochemistry to test for the presence of CXCL12 expression in a series of primary 47 tongue OSCC specimens. In 30 (63.8%) of the specimens, cancer cells were positive for CXCL12 staining. When samples were divided based on histological grade, 11 of 13 grade 1 tumors (84.6%), 17 of 26 grade 2 tumors (65.4%), and 2 of 8 grade 3 tumors (25.0%) contained CXCL12‐positive cancer cells (*p* = 0.021). Expression of CXCL12 in tumor cells correlated positively with the degree of keratinization, whereas it correlated negatively with histological grade (Figure 2A, B). Grade 1 tumors without invasion of the muscularis propria had high neoplastic cell content, and a majority of the tumor area was positive for CXCL12 (Figure 2A, Figure S1A). By contrast, CXCL12‐positive areas were smaller in higher grade tumors exhibiting muscle invasion (Figure 2B, Figure S1B, *p* = 0.047).

We next focused on CXCL12 expression in stromal cells. In non‐tumorous tongue tissues adjacent to tumors, CXCL12 was expressed in both muscle and endothelial cells (Figure 2A, B). Within invasive front regions, we noted that grade 1 tumors showed higher levels of stromal CXCL12 expression than did grade 3 tumors (Figure 2A, B). At the centers of grade 1 tumors, muscle cells retained a normal appearance and CXCL12 expression, while muscle cells at the center of grade 3 tumors exhibited disrupted and atrophic structures, which led to reductions in the CXCL12‐positive areas (Figure 2A, B). Quantitative analysis of muscle cell areas also confirmed significant reduction of muscle cells at the center of grade 3 tumors (Figure S2).

When tumors were divided according to their histological grade and T category, we found that areas positive for CXCL12 were larger in grade 1 than grade 3 tumors, irrespective of the T category (Figure 2C). Among grade 2 tumors, by contrast, those with greater T category exhibited higher levels of CXCL12 expression than T1 tumors (Figure 2C). The correlations between CXCL12 expression and clinicopathological characteristics of the OSCC samples are summarized in Table 1. Lower levels of CXCL12 expression were positively correlated with poor differentiation, mode of invasion, and tumor recurrence (Table 1, Figure 2D). Finally, Kaplan–Meier curve analysis demonstrated that lower CXCL12 expression was associated with poorer overall survival among the patients (Figure 2E). These results suggest that reduced stromal CXCL12 expression is positively associated with the aggressiveness of OSCC.

**TABLE 1:** CXCL12 expression and clinicopathological characteristics of tongue OSCC

	CXCL12‐low	CXCL12‐high	*p*
Age			
<65	11	13	0.773
≥65	12	11	
Gender			
Male	16	19	0.517
Female	7	5	
Tumor size			
T1 or T2	14	17	0.547
T3 or T4	9	7	
Lymph node metastasis			
Positive	5	5	1
Negative	18	19	
Stage			
I or II	14	17	0.547
III or IV	9	7	
Differentiation			
Well or moderate	16	23	0.023
Poor	7	1	
Mode of invasion			
YK‐2 or 3	11	20	0.015
YK‐4C or 4D	12	4	
Recurrence			
Yes	13	6	0.039
No	10	18	

Abbreviation: OSCC, oral squamous cell carcinoma.

### 3.3 CXCL12 expression correlates with α‐SMA expression and CD8+ T‐cell infiltration

**FIGURE 3:**
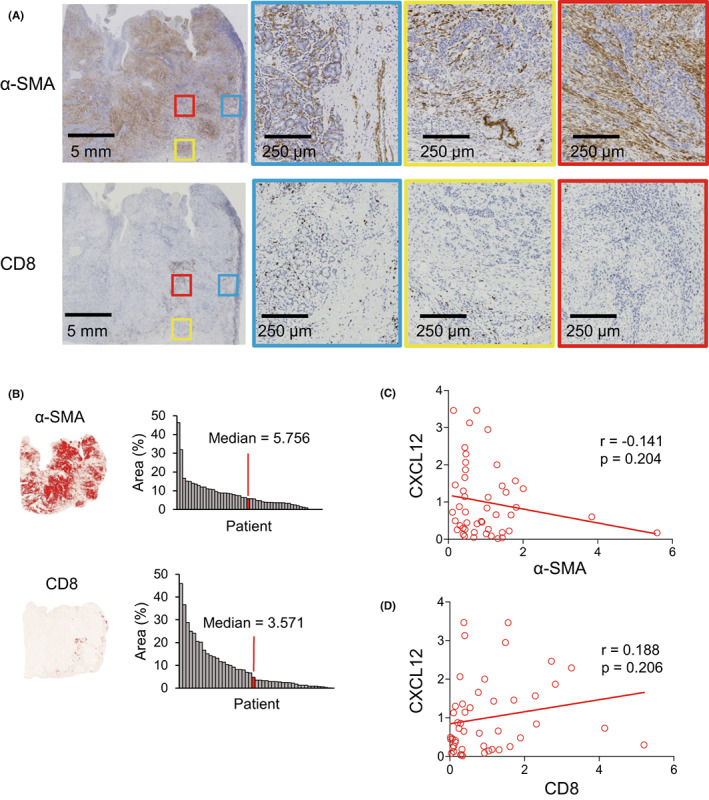
Comparison of CXCL12 expression with expression of stromal markers and CD8‐positive lymphocyte infiltration in OSCC. (A) Immunohistochemical detection of α‐SMA and CD8 in representative OSCC tissues. Magnified views of adjacent normal tissues (blue), invasive front regions (yellow), and central tumor areas (red) are also shown. (B) Areas positive for the indicated markers in representative tumors are shown on the left. Summarized results for areas positive for the indicated markers in OSCC tissues (*n* = 47) are shown on the right. (C, D) Correlations between areas positive for CXCL12 and those positive for α‐SMA (C) or CD8 (D).

The results summarized above suggest that CXCL12 is expressed in both tumor and stromal cells within OSCC tissues and that its expression is inversely associated with the clinical aggressiveness of the tumor. To more precisely determine the distribution of CXCL12 expression within the tumor microenvironment, we used immunohistochemistry to assess the relationship between CXCL12 expression and expression of the CAF marker α‐SMA within OSCC specimens (Figure 3A). Quantitative analysis of the areas with positive staining revealed that levels of α‐SMA expression tended to correlate inversely with those of CXCL12, though we could not make definitive conclusions due to the p‐value (Figure 3B, C).

Recent studies also showed that tumor microenvironment plays an essential role in tumor immunology. We therefore assessed infiltration by CD8+ T lymphocytes into OSCC tissues. Notably, we observed that the levels of CXCL12 expression tended to correlate positively with infiltration by CD8+ cells, though we could not make definitive conclusions due to the p‐value (Figure 3B, D).

### 3.4 OSCC cells upregulate CXCL12 expression in muscle cells

**FIGURE 4:**
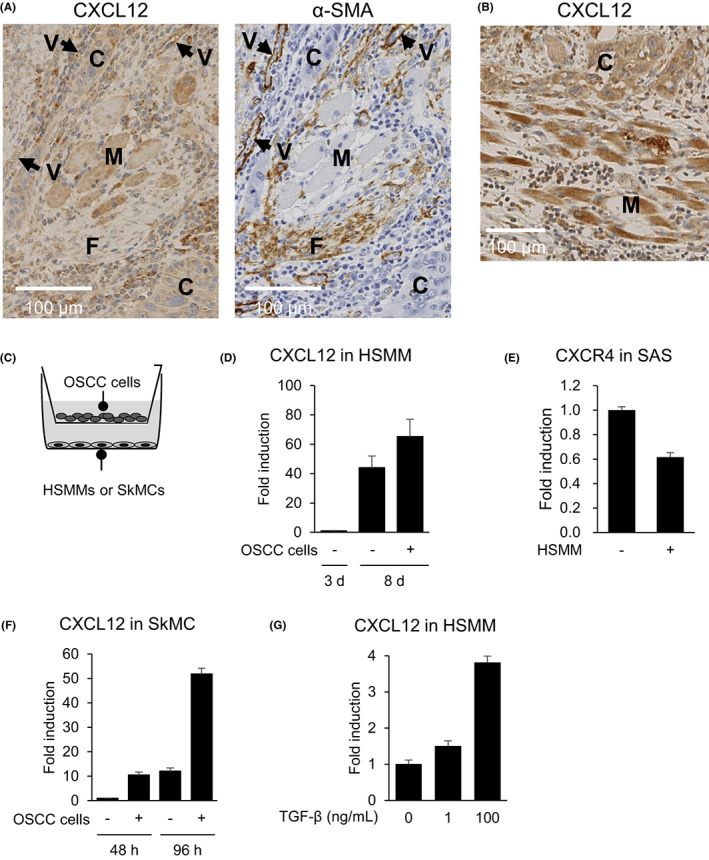
CXCL12 expression in muscle cells is upregulated by OSCC cells. (A) Immunohistochemical detection of CXCL12 (left) and α‐SMA (right) in representative OSCCs. C, cancer cells; M, muscle cells; F, fibroblasts; V, vessels. (B) Immunohistochemical detection of CXCL12 in representative OSCC tissue. C, cancer cells; M, muscle cells. (C) Schematic representation of the co‐culture experiments. Human skeletal muscle myoblasts (HSMMs) or skeletal muscle cells (SkMCs) were indirectly co‐cultured with the SAS OSCC cell line. (D) qRT‐PCR analysis of CXCL12 in HSMMs co‐cultured with or without SAS cells for the indicated times. Shown are means of 3 replications; error bars represent SDs. (E) qRT‐PCR of CXCR4 in SAS cells co‐cultured with or without HSMMs for 8 days. Shown are means of 3 replications; error bars represent SDs. (F) qRT‐PCR analysis of CXCL12 in SkMCs co‐cultured with or without SAS cells for the indicated times. Shown are means of 3 replications; error bars represent SDs. (G) qRT‐PCR of CXCL12 in HSMMs treated with indicated concentrations of TGF‐β1. Shown are means of 3 replications; error bars represent SDs. OSCC, oral squamous cell carcinoma; qRT‐PCR, quantitative reverse transcription.

The single‐cell RNA‐seq data suggested that CXCL12 is preferentially expressed in fibroblasts within HNSCCs. However, we noted that the levels of CXCL12 expression were lower in fibroblasts within tongue OSCC tissues than in neighboring muscle cells (Figure 4A). Moreover, muscle cells adjacent to tumor cells often exhibited abundant CXCL12 expression (Figure 4B). We therefore performed co‐culture experiments to test whether OSCC cells stimulate upregulation of CXCL12 in nearby muscle cells (Figure 4C). We observed that differentiation of HSMM cells into muscle cells induced by culture for 8 days in skeletal muscle growth medium significantly upregulated CXCL12 expression (Figure 4D). When HSMM cells were indirectly co‐cultured with OSCC cells for 8 days, the expression of CXCL12 was further upregulated (Figure 4D). By contrast, levels of CXCR4 expression were not elevated in OSCC cells co‐cultured for 8 days with or without HSMMs; instead, they were somewhat decreased in OSCC cells co‐cultured with HSMMs (Figure 4E). In addition, we found that indirect co‐culture with OSCC cells upregulated CXCL12 in skeletal muscle cells, and longer exposure to OSCC cells led to higher levels of CXCL12 expression (Figure 4F).

Recent studies showed that TGF‐β regulates CXCL12 expression in several types of tumors other than HNSCC.^20^, ^21^ We therefore treated HSMM cells with TGF‐β1 and found that CXCL12 was upregulated in a dose‐dependent manner (Figure 4G). This suggests OSCC cells may upregulate CXCL12 expression, at least in part, through TGF‐β signaling.

## 4 DISCUSSION

In this study, we found that CXCL12 is expressed in tumor, stromal, and muscle cells within primary OSCC tissues. We also demonstrated that higher levels of CXCL12 expression are associated with a better prognosis in OSCC patients.

Grade 1 tumors were characterized by focal tumor cell proliferation, collective migration, and greater degrees of keratinization, as well as abundant CXCL12 expression. By contrast, grade 3 tumors showed disseminating single cell invasion into the muscularis propria, which caused disruption of the muscle structure and a decrease in muscle cell numbers and eventually led to a reduction in CXCL12‐positive areas within the tumor tissues. Interestingly, in grade 2 tumors, greater T category was strongly associated with higher levels of CXCL12 expression. Taken together with the results of our co‐culture experiments, these observations suggest that tumor cell invasion into muscle tissues may lead to CXCL12 upregulation in muscle cells.

Because single‐cell RNA‐seq results suggested that fibroblasts are a major source of CXCL12 expression in HNSCC, we compared the immunohistochemical findings for CXCL12 with those for α‐SMA in OSCC tissues.^14^ Unexpectedly, we found that CXCL12‐positive areas tended to be inversely correlated with α‐SMA‐positive areas, though the relation was not statistically significant. This is consistent with our finding that CXCL12 is expressed in cancer cells and muscle cells but is less abundantly expressed in fibroblasts. We also found a tendency for CXCL12 expression to correlate positively with CD8+ T‐cell infiltration. Although the relation was not statistically significant, it may be consistent with the favorable outcomes of OSCC patients exhibiting higher CXCL12 expression. Recent studies suggest that CXCL12 derived from tumor stromal cells contributes to tumor progression, but our results are contradictory to those observations.^2^, ^4^

One explanation for that discrepancy is related to our finding that muscle cells within OSCC tissues are a novel source of CXCL12 expression. Zhang et al. reported that CXCL12 expression in cancer cells was observed in the majority (68.0%) of OSCCs, which is consistent with our present results.^12^ In addition, we also found that CXCL12 is expressed in muscle cells adjacent to cancer cells within tongue OSCC tissues. We confirmed experimentally that OSCC cells upregulate CXCL12 expression in skeletal muscle cells. Notably, we also observed that the disruption of muscle tissue structure in grade 3 tumors is associated with a reduction in CXCL12‐positive areas within the tumor. These findings suggest that early during tongue OSCC development, OSCC cells stimulate muscle cells to express CXCL12, which may contribute to tumor progression. However, destruction of the muscle tissues in the advanced stages leads to a decrease in overall tumoral CXCL12 expression.

Anatomically, invasion of the tongue by OSCCs leads to interaction between cancer cells and the skeletal muscles of the tongue, though the role of muscle cells in the tumor microenvironment is not fully understood. In normal tissues, the CXCL12/CXCR4 axis plays an important role in skeletal muscle development and regeneration.^22^, ^23^, ^24^, ^25^ It is therefore possible that CXCL12 secreted by muscle cells may functionally affect OSCC cells, though it remains unclear whether the muscle cell‐derived CXCL12 contributes to or inhibits tumor progression.

In this study, we found that OSCC cells upregulate CXCL12 in muscle cells, at least in part, via TGF‐β signaling. Similarly, a recent study showed that CXCL12‐CXCR4 signaling triggered by TGF‐β contributes to drug resistance in HNSCC cells.^20^ Another study showed that TGF‐β secreted by endometrial cancer cells induces CXCL12 production by endometrial mesenchymal stem cells.^21^ By contrast, other studies reported that TGF‐β suppresses CXCL12 expression in both bone marrow and mesenchymal stromal cells.^26^, ^27^

There are several limitations to this study. First, although our results suggest that OSCC cell‐derived factors upregulate CXCL12 expression in muscle cells, the underlying mechanism is not fully understood. A more comprehensive analysis will be necessary to identify cancer cell‐derived factors that induce CXCL12 expression in muscle cells. Second, as described above, the biological function of CXCL12 secreted by muscle cells in OSCC tissues remains unknown. If muscle cell‐derived CXCL12 contributes to the development of OSCC, it appears paradoxical that expression of CXCL12 is reduced in advanced stage tumors. As we did not observe upregulation of CXCR4 in OSCC cells co‐cultured with muscle cells, it remains unclear whether interaction between OSCC cells and muscle cells can activate CXCL12‐CXCR4 signaling. Further studies will be needed to unravel the biological and clinical significance of muscle cell‐derived CXCL12 in tongue OSCCs.

In summary, we found that skeletal muscle cells within tongue OSCC tissues express CSCL12, and lower tumoral CXCL12 expression is associated with a poorer prognosis. The interaction between tongue skeletal muscle cells and tumor cells appears to impact tumor progression, but the molecular mechanisms remain unclear. Further study is needed to fully understand the tumor microenvironment in OSCC.

## AUTHOR CONTRIBUTIONS

**Akira Yorozu:** Conceptualization (equal); data curation (lead); funding acquisition (equal); investigation (equal); writing – original draft (equal); writing – review and editing (equal). **Shohei Sekiguchi:** Data curation (supporting); investigation (equal); writing – review and editing (supporting). **Akira Takasawa:** Data curation (supporting); investigation (supporting); writing – review and editing (supporting). **Fumika Okazaki:** Data curation (supporting); investigation (supporting); writing – review and editing (supporting). **Takeshi Niinuma:** Funding acquisition (supporting); investigation (supporting); writing – review and editing (supporting). **Hiroshi Kitajima:** Investigation (supporting); writing – review and editing (supporting). **Eiichiro Yamamoto:** Funding acquisition (supporting); writing – review and editing (supporting). **Masahiro Kai:** Investigation (supporting); writing – review and editing (supporting). **Mutsumi Toyota:** Investigation (supporting); writing – review and editing (supporting). **Yui Hatanaka:** Investigation (supporting); writing – review and editing (supporting). **Koyo Nishiyama:** Resources (supporting); writing – review and editing (supporting). **Kazuhiro Ogi:** Resources (supporting); writing – review and editing (supporting). **Hironari Dehari:** Resources (supporting); writing – review and editing (supporting). **Kazufumi Obata:** Resources (supporting); writing – review and editing (supporting). **Makoto Kurose:** Resources (supporting); writing – review and editing (supporting). **Atsushi Kondo:** Resources (supporting); writing – review and editing (supporting). **Makoto Osanai:** Investigation (supporting); writing – review and editing (supporting). **Akihiro Miyazaki:** Resources (supporting); supervision (equal); writing – review and editing (supporting). **Kenichi Takano:** Supervision (equal); writing – review and editing (supporting). **Hiromu Suzuki:** Conceptualization (equal); funding acquisition (equal); project administration (lead); writing – original draft (equal); writing – review and editing (equal).

## CONFLICT OF INTEREST

All authors declare no conflict of interest.

## ETHICS APPROVAL

This study was approved by the Institutional Review Board at Sapporo Medical University (No. 322–38).

## Supporting information


Figures S1–S2

## Data Availability

The datasets used and analyzed during the current study are available from the corresponding author upon reasonable request.
