# Supplementary material for: CXCL12 is expressed by skeletal muscle cells in tongue oral squamous cell carcinoma
Source: Cancer Med. 2022 Oct 27;12(5):5953–63. doi: 10.1002/cam4.5392 (PMC10028106; doi:10.1002/cam4.5392)
Supplement: Supplementary file 1 — Figures S1–S2 [file CAM4-12-5953-s001.docx]

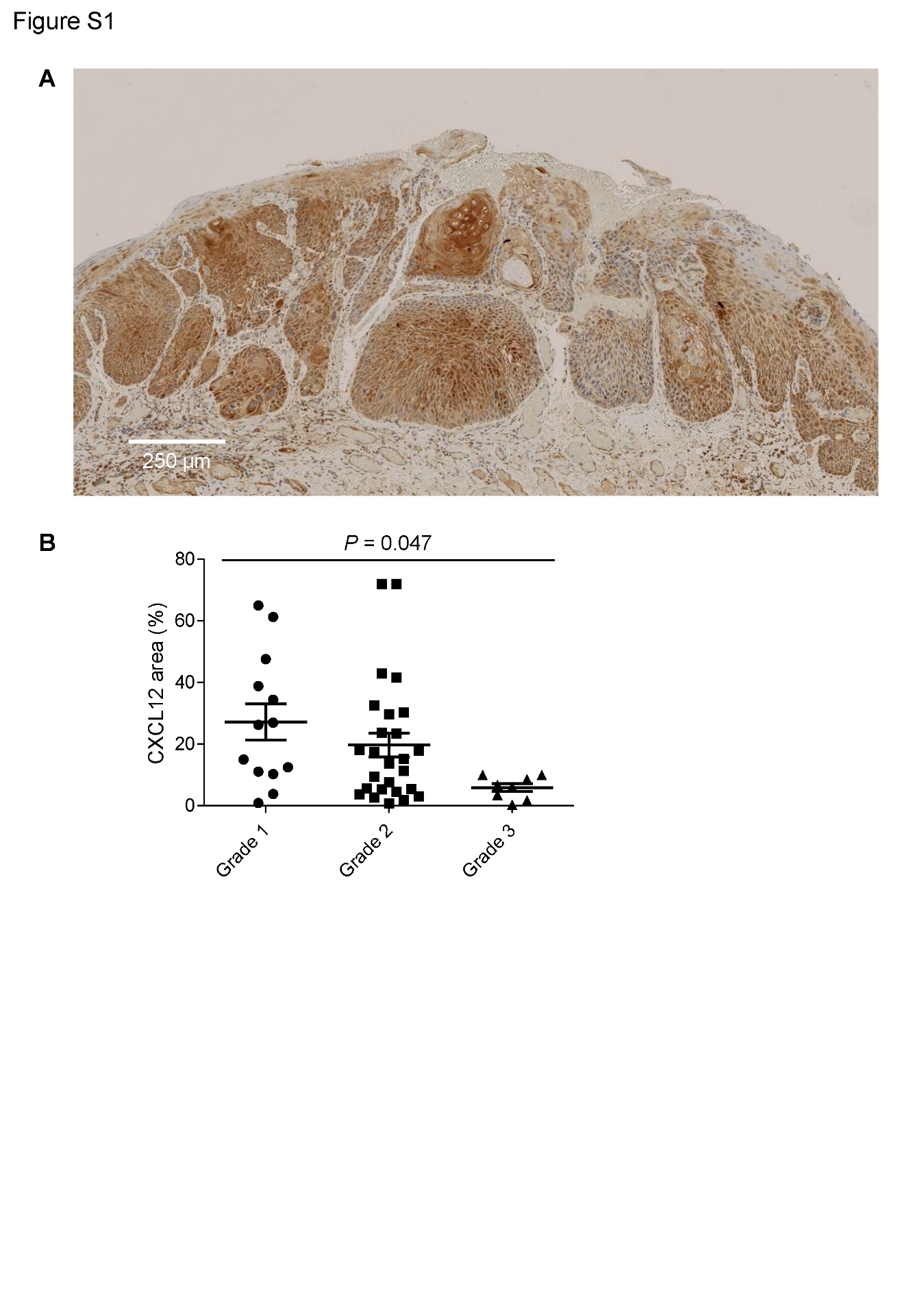


**Figure S1**

Analysis of CXCL12 expression in primary OSCC tissues. (A) Immunohistochemical detection of CXCL12 in a representative grade 1 OSCC tissue specimen without invasion of the muscularis propria. (B) The extents of CXCL12-positive areas within OSCC tissues with indicated staging.


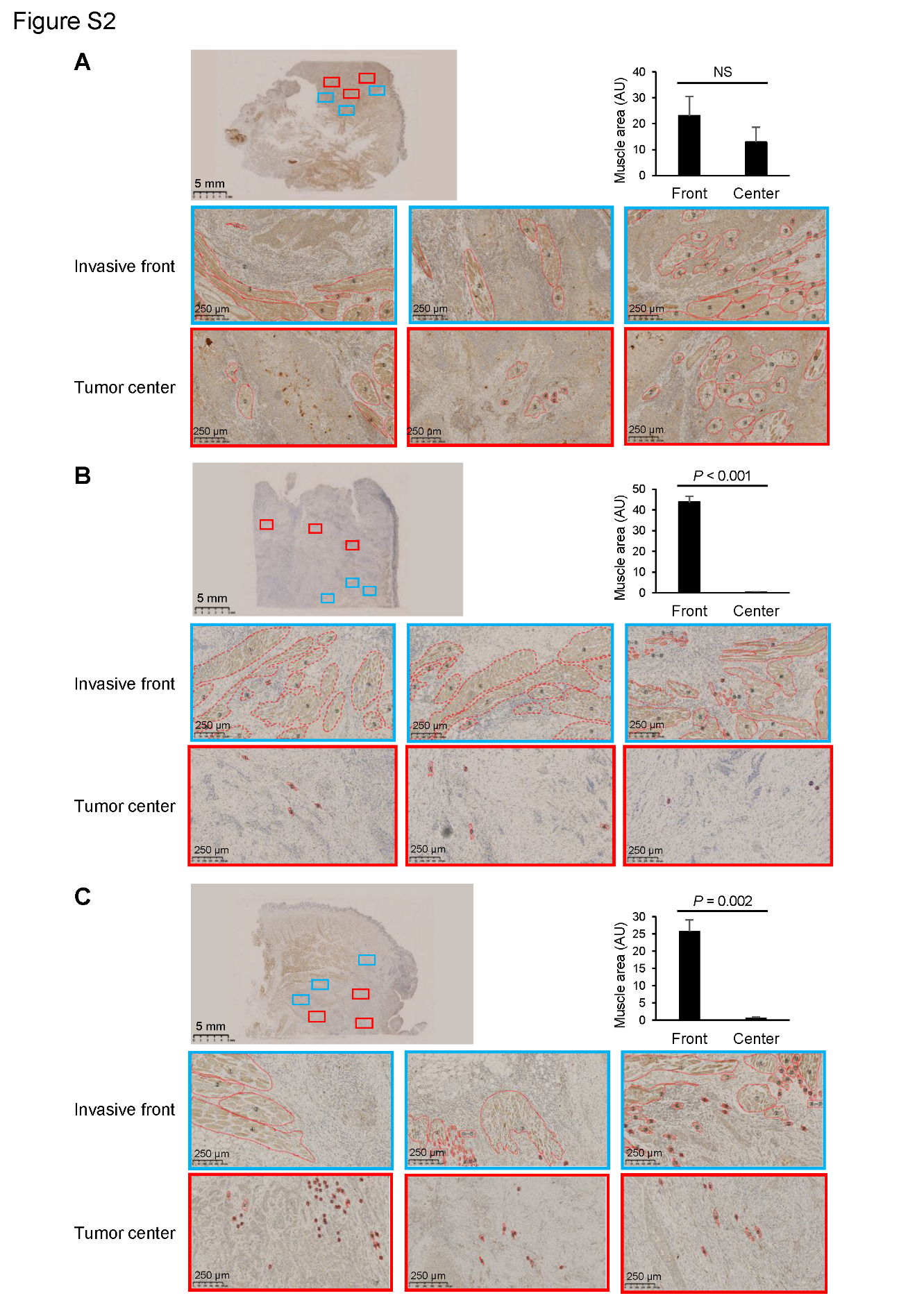


**Figure S2**

Quantitative analysis of muscle cell areas in primary OSCC tissues. Three representative areas were selected from invasive front (blue) and central regions (red) in a grade 1 tumor (A) and grade 3 tumors (B and C) with CXCL12 staining. Muscle cell areas were selected and quantified using ImageJ software. Summarized results of muscle cell areas in indicated areas in respective tumors are shown on the upper right.
